# Midwives

**Published:** 1879-11

**Authors:** 


### EDITORIAL.

---

### MIDWIVES.

THE subject of midwives, in its relations to the medical profession and the public, is one of so great importance that we have no apology to offer in presenting it for the consideration of the readers of the JOURNAL. The position of the midwife is one of great responsibility. Admitting that many times parturition is a purely physiological process, even here meddlesome midwifery is capable of doing much harm; and it is generally admitted that ignorance and officiousness go hand-in-hand. Considering the great responsibility of the midwife when in the discharge of her duties, one, unacquainted with the facts, would naturally presume that there must exist sufficient legal safeguards prohibiting any, but those well qualified, from assuming the duties of the accoucheur. This, however, is quite far from the truth.

Prior to 1874, there was practically no law upon the subject. In the spring of the year mentioned the Legislature, then in session, enacted the following law:

### CHAPTER 436.

### AN ACT TO REGULATE THE PRACTICE OF MEDICINE AND SURGERY IN THE STATE OF NEW YORK.

Passed May 11, 1874.

*The People of the State of New York, represented in Senate and Assembly, do enact as follows:*

SECTION 1. Every practitioner of medicine or surgery in this State, excepting licentiates or graduates of some medical society or chartered school, shall be required, and they are hereby commanded, to obtain a certificate from the censors of some one of the several medical societies of this State, either from the county, district or State society; which certificate shall set forth that said censors have found the person to whom it was issued qualified to practice all the branches of the medical art mentioned in it. And such certificate must be recorded in a book provided and kept for the purpose by the County Clerk of each county in the State.

§ 2. The censors of each medical society aforesaid shall notify all practitioners of medicine and surgery of the terms and requirements of this act, and shall request such persons, so notified, to comply with those requirements within thirty days after such notification; and if such persons shall not, within the time specified in the notice, or within such further time as may be allowed by special arrangement with said censors, not exceeding ninety days, comply with the requirements herein made of physicians or surgeons, as the case may be, such persons shall thereafter be subject to all the provisions and penalties prescribed by this act for any violation of the same, and the president of the society making such request shall and he is hereby required to at once commence the proceedings authorized by this act against such person.

§ 3. It is hereby declared a misdemeanor for any person to practice medicine or surgery in this State, unless authorized so to do by a license or diploma from some chartered school, State board of medical examiners, or medical society, or who shall practice under cover of a medical diploma illegally obtained; and any person found guilty of such a misdemeanor shall, for the first offense, be fined not less than fifty nor more than two hundred dollars; for any subsequent offense, not less than one hundred nor more than five hundred dollars, or by imprisonment not less than thirty days, or by both imprisonment and fine; and all such fines shall go into the county treasury of the county bringing such action.

Assuming that a midwife is a practitioner of medicine or surgery, the law, as it now stands, would appear to be quite equal to the requirements of the case. This, however, has not proved to be so. Shortly after the above law became a part of the statute, the censors of the Medical Society of the County of Erie notified a number of irregular practitioners, and soon supplemented this by commencing legal proceedings against the parties. These proceedings, in accordance with legal advice, were almost immediately discontinued; the opinion being that, under the loose reading of the present law, a conviction could not be expected. So far as we are aware nothing has since been done or attempted.

That the average midwife is entirely unqualified for the intelligent discharge of her duties goes without saying. We have in the course of our practice met with many forcible illustrations. Time and time again, when we have been called to rectify a supposed cross-birth, it has proved to be a head presentation. Again we have been sent for to apply the forceps when the os was not perceptibly dilated, and real labor did not come on for a number of days. As we all know, these illustrations might be multiplied almost without end. Nor is this to be wondered at. It is the natural result of the state of things which allows any woman, whose privilege it has been to witness a half-dozen confinements, to assume the duties and responsibilities of an attendant to the parturient. Many, many lives are annually lost as a result of this ignorance, and many more rendered permanently miserable.

In conclusion, as a remedy for these evils we would suggest first, that physicians once more unite in the effort to pass a bill which shall be so worded, and otherwise sufficient, that under it convictions may be procured; and secondly, that schools be instituted for the instruction of those desiring to become midwives. For this a model might be obtained in the present schools for nurses throughout the country, and of which we have one in this city, in connection with the Buffalo General Hospital. That such schools are practicable is seen in the fact that similar schools have long been in existence in Germany and other European countries. In connection with the various Charity Organizations, no difficulty would be found in providing means for practical instruction and study. With schools of this kind, issuing diplomas certifying to the qualifications of the graduates, there would no longer be an excuse for the imposition now practiced.

